# Acute-on-chronic liver failure syndrome - clinical results from an intensive care unit in a liver transplant center

**DOI:** 10.5935/0103-507X.20200009

**Published:** 2020

**Authors:** Rui Pereira, Luís Bagulho, Filipe Sousa Cardoso

**Affiliations:** 1 Intensive Care Unit, Hospital Curry Cabral, Centro Hospitalar Universitário Lisboa Central - Lisboa, Portugal.; 2 Transplant Unit, Hospital Curry Cabral, Centro Hospitalar Universitário Lisboa Central - Lisboa, Portugal.

**Keywords:** Cirrhosis, Critical illness, Multiple organ failure, Lactate, International normalized ratio, Treatment outcome, Cirrose, Doença crítica, Falência múltipla dos órgãos, Lactato, Coeficiente internacional normalizado, Resultado do tratamento

## Abstract

**Objective:**

To characterize a cohort of acute-on-chronic liver failure patients in Intensive Care and to analyze the all-cause 28-day mortality risk factors assessed at ICU admission and day 3.

**Methods:**

This was a retrospective cohort study of consecutive patients admitted to the intensive care unit between March 2013 and December 2016.

**Results:**

Seventy-one patients were included. The median age was 59 (51 - 64) years, and 81.7% of patients were male. Alcohol consumption alone (53.5%) was the most frequent etiology of cirrhosis and infection (53.5%) was the most common acute-on-chronic liver failure precipitating event. At intensive care unit admission, the clinical severity scores were APACHE II 21 (16 - 23), CLIF-SOFA 13 (11 - 15), Child-Pugh 12 (10 - 13) and MELD 27 (20 - 32). The acute-on-chronic liver failure scores were no-acute-on-chronic liver failure: 11.3%; one: 14.1%; two: 28.2% and three: 46.5%; and the number of organ failures was one: 4.2%; two: 42.3%; three: 32.4%; four: 16.9%; and five: 4.2%. Liver transplantation was performed in 15.5% of patients. The twenty-eight-day mortality rate was 56.3%, and the in-ICU mortality rate was 49.3%. Organ failure at intensive care unit admission (p = 0.02; OR 2.1; 95%CI 1.2 - 3.9), lactate concentration on day 3 (p = 0.02; OR 6.3; 95%CI 1.4 - 28.6) and the international normalized ratio on day 3 (p = 0.03; OR 10.2; 95%CI 1.3 - 82.8) were independent risk factors.

**Conclusion:**

Acute-on-chronic liver failure patients presented with high clinical severity and mortality rates. The number of organ failures at intensive care unit admission and the lactate and international normalized ratio on day 3 were independent risk factors for 28-day mortality. We consider intensive care essential for acute-on-chronic liver failure patients and timely liver transplant was vital for selected patients.

## Abstract (pt)

**Objetivo:**

Caracterizar uma coorte de pacientes com insuficiência hepática crônica agudizada em unidade de terapia intensiva e analisar os fatores de risco da mortalidade global aos 28 dias presentes no dia da admissão e no dia 3.

**Métodos:**

Estudo de coorte retrospectiva de pacientes consecutivamente admitidos à unidade de terapia intensiva entre março de 2013 e dezembro de 2016.

**Resultados:**

Incluímos 71 pacientes com idade mediana de 59 (51 - 64) anos, sendo 81,7% do sexo masculino. Consumo de álcool isoladamente (53,5%) foi a etiologia mais frequente de cirrose, e infecção (53,5%) foi o evento que mais frequentemente precipitou a agudização da insuficiência hepática crônica. No momento da admissão à unidade de terapia intensiva, os escores de severidade foram: APACHE II 21 (16 - 23), CLIF-SOFA 13 (11 - 15), Child-Pugh 12 (10 - 13) e MELD 27 (20 - 32). Dentre os indivíduos, 14,1% tiveram escore 1 de insuficiência hepática crônica agudizada, 28,2% pontuaram 2, 64,5% pontuaram 3, e 11,3% se apresentaram sem insuficiência hepática crônica agudizada. Sobre o número de falências de órgãos, 4,2% tiveram um órgão; 42,3%, dois órgãos; 32,4%, três órgãos; 16,9%, quatro órgãos; e 4,2% cinco órgãos. Foi realizado transplante hepático em 15,5% dos pacientes. A taxa de mortalidade aos 28 dias foi de 56,3%, e a taxa de mortalidade na unidade de terapia intensiva foi de 49,3%. Foram fatores independentes de risco: falências do órgãos quando da admissão à unidade de terapia intensiva (p = 0,02; RC 2,1; IC95% 1,2 - 3,9), concentração de lactato em sangue arterial no dia 3 (p = 0,02; RC 6,3; IC95% 1,4 - 28,6) e RC internacional no dia 3 (p = 0,03; RC 10,2; IC95% 1,3 - 82,8).

**Conclusão:**

Pacientes com insuficiência hepática crônica agudizada apresentaram elevados níveis de severidade e taxa de mortalidade. O número de falências de órgãos no momento da admissão à unidade de terapia intensiva e o nível de lactato, assim como a razão normalizada internacional no dia 3 de permanência na unidade foram fatores independentes de risco para a mortalidade ao 28º dia. Consideramos que a terapia intensiva é essencial para pacientes com insuficiência hepática crônica agudizada, e a realização de um transplante hepático em tempo adequado foi vital para os pacientes selecionados.

## INTRODUCTION

The critically ill cirrhotic patient poses a challenge for intensive care specialists. Therapeutic advances such as albumin dialysis, high volume plasma exchange and transplantation in increasingly severe patients have brought critically ill cirrhotic patients closer to the intensive care setting. Nonetheless, high mortality rates and advanced chronic liver disease, combined with acute critical illness, represent increased difficulty in determining which cirrhotic patients benefit most from intensive care therapy.^(1-4)^

Chronic liver disease patients hospitalized with acute decompensation can develop an inflammatory state with ensuing acute organ failure leading to acute-on-chronic liver failure (ACLF) syndrome.^(5-7)^ Acute-on-chronic liver failure syndrome, well characterized in the multicentric CANONIC study, represents a severe subgroup of cirrhotic patients with acute organ failure as assessed by the Chronic Liver Failure - Sequential Organ Failure Assessment (CLIF-SOFA) score.^(6)^ These patients present a complex clinical course with high short-term mortality rates despite vital organ support.^(8-10)^ Liver transplantation (LT) may be an option for some patients, although the selection of potential candidates and the timing for this intervention remains complex.^(3,11)^ Ethical issues concerning futility of care while receiving vital organ support in the intensive care unit (ICU) increase the complexity of the clinical management of these patients.^(4,12-14)^

We present epidemiologic data from a single center cohort of ACLF patients admitted to a general ICU in a regional Portuguese liver transplant center.

The primary aim of this study was to determine the all-cause 28-day mortality rate post-ICU admission, and the secondary aim was to analyze 28-day mortality risk factors assessed at ICU admission (day 0) and 72 hours (day 3) thereafter.

## METHODS

This was a retrospective analysis of a prospective registry including consecutive patients with ACLF in a general ICU integrated in a regional reference center for LT at *Hospital Curry Cabral*, Lisboa, Portugal, between 03/2013 and 12/2016.

The inclusion criteria were as follows: age ≥18 years; previous diagnosis of cirrhosis, acute organ failure present or expected at ICU admission, and first ICU admission from the index hospital stay; and the exclusion criteria were as follows: acute liver failure, cirrhosis without organ failure during the ICU stay; history of previous LT; and ICU admission for surgical reasons.

All data on patient characteristics were retrieved from medical records and collected in an anonymous and protected database. The clinical data and variables were collected at ICU admission (day 0) and after 72 hours (day 3) for 28-day mortality risk factor analysis. The following clinical variables were assessed: age, sex, etiology (alcohol, hepatocellular carcinoma - HCC), previous decompensation and complications (ascites, portal vein thrombosis and HCC) of cirrhosis, precipitating event of acute illness (alcohol, infection, bleeding, and others), general laboratory parameters including complete hemogram, biochemical serum analyses and arterial blood lactate concentration, clinical severity scores (Acute Physiology and Chronic Health Evaluation II - APACHE II at day 0 and CLIF-SOFA, Delta CLIF-SOFA, ACLF classification, Child-Pugh and Model for End-Stage Liver Disease - MELD scores at day 0 and day 3) and organ support (vasopressors, mechanical ventilation, and renal replacement therapy).

Organ failure in this study used the CLIF-SOFA score definition. The calculation of the Delta CLIF-SOFA used day 3 minus day 0 scores.

Bleeding-a category of the “precipitating event” variable-was transformed into a dichotomous variable for multivariate logistic regression.

### Statistical analysis

Continuous and categorical variables were described as medians (interquartile ranges - IQRs) and frequencies (percentages - %), respectively. The univariate analysis of 28-day mortality rate risk associations was performed using Mann-Whitney and Chi-square tests, where appropriate, for day 0 and day 3 variables. The multivariate backward stepwise logistic regression included day 0 and day 3 variables with p < 0.10 on univariate analysis and excluded variables with p > 0,10 from the final model. Survival analysis used the Kaplan-Meier method. Statistical significance was defined as p ≤ 0.05. Statistical analysis was performed using IBM Statistical Package for Social Science (SPSS), version 23 (IBM Corp, North Castle, NY, US).

### Definitions, exposures and outcomes

Cirrhosis was defined as bridging fibrosis on previous liver biopsy or a composite of clinical signs and findings provided by laboratory tests, endoscopy, and radiologic imaging.^(5)^ Despite worldwide variations in the definition of ACLF, we defined organ failures and ACLF as per the European Foundation for the Study of Chronic Liver Failure Consortium (CLIF-C) definitions.^(6)^

The general ICU and specific cirrhosis clinical severity of illness scales considered on ICU admission and at Day 3 were the following: the CLIF-SOFA score and ACLF grading system, the Child-Pugh score, the MELD score, and the APACHE II score.

The CLIF-SOFA score is a validated adaptation of the classical Sequential Organ Failure Assessment (SOFA) score to assess the severity of disease in patients with cirrhosis. It comprises the grading of 6 different organ systems (each ranging from 0 [least severe] to 4 [most severe] and overall from 0 to 24): brain, cardiovascular system, lungs, kidneys, liver, and coagulation.^(6)^ This study used the definitions of organ failure as *per* CLIF-SOFA.

The ACLF grading system ranks patients with ACLF in one of 3 grades based on the number of failing organs according to the CLIF-SOFA score: grade 1 if kidney failure (serum creatinine ≥ 2.0mg/dL) or other any other single organ failure with serum creatinine 1.5 - 1.9mg/dL or West Haven grade I-II hepatic encephalopathy present, grade 2 if 2 organ failures are diagnosed, and grade 3 if 3 or more organ failures have developed.^(6)^

The Child-Pugh score was originally developed to predict the 6-month mortality of patients with cirrhosis who underwent esophageal surgical ligation for bleeding varices but is now generally used to determine the 6-month mortality risk for all patients with complicated cirrhosis. It comprises the assessment of the following characteristics (ranging each from 1 [least severe] to 3 [most severe] and overall from 5 to 15): international normalized ratio (INR), bilirubin, albumin, hepatic encephalopathy, and ascites.^(15)^

The MELD score was initially developed to predict 3-month mortality for patients with cirrhosis and portal hypertension who underwent a transjugular intrahepatic portal shunt procedure, but it is now widely used to assess the 3-month mortality risk of these patients while on the waitlist for LT. It is based on the evaluation of liver (INR and bilirubin) and renal (creatinine) function.^(16)^

The APACHE II score has been widely used for several decades to assess the severity of disease in general patients in the ICU. It is based on 12 routine physiologic measurements, age, previous health status (chronic disease and/or immunodeficiency), and surgical status to predict the risk of hospital mortality (overall score ranges from 0 to 71).^(17)^

The Institutional Ethics Committee waived the need for individual informed consent for this noninterventional study. All study procedures followed the principles of the Declaration of Helsinki. The reporting of this study followed the Strengthening the Reporting of Observational Studies in Epidemiology (STROBE) guidelines.

## RESULTS

All patient characteristics and respective univariate 28-day mortality rates at ICU admission and at day 3 post-ICU admission are detailed in tables 1 and 2, respectively.

**Table 1:** Acute-on-chronic liver failure patient characteristics at intensive care unit admission (day 0) and univariate association with 28-day mortality

Characteristics	Overall (n = 71)	Dead at 28 days (n = 40)	Alive at 28 days (n = 31)	p value	OR	OR 95%CI	
						Inferior	Superior
Age	59 (51 - 64)	60 (50 - 65)	59 (51 - 63)	0.29	1.0	1.0	1.0
Sex (male)	58 (81.7)	32 (80.0)	26 (83.9)	0.41	1.6	0.5	4.9
Etiology							
Alcohol	38 (53.5)	22 (55.0)	16 (51.6)	0.67	ref.		
Hepatitis C virus	7 (9.9)	4 (10.0)	3 (9.7)	0.33	1.4	0.7	2.6
Alcohol + hepatitis C virus	8 (11.3)	3 (7.5)	5 (16.1)	0.71	1.3	0.3	6.0
Other	18 (25.4)	11 (27.5)	7 (22.6)	0.48	0.6	0.1	2.5
Previous decompensation	61 (85.9)	35 (87.5)	26 (83.9)	1.00	1.0	0.3	3.5
Ascites	64 (90.1)	36 (90.0)	28 (90.3)	0.71	1.3	0.3	6.0
Hepatocellular carcinoma	7 (9.9)	4 (10.0)	3 (9.7)	0.32	1.3	0.8	2.1
Portal vein thrombosis	7 (9.9)	5 (12.5)	2 (6.5)	0.45	1.2	0.7	2.0
Precipitating event							
Alcohol	5 (7.0)	2 (5.0)	3 (9.7)	0.17	Reference		
Infection	38 (53.5)	26 (65.0)	12 (38.7)	0.66	0.7	0.1	4.0
Bleeding	16 (22.5)	6 (15.0)	10 (32.3)	0.03	2.2	1.1	4.3
Other	3 (4.2)	2 (5.0)	1 (3.2)	0.32	0.6	0.2	1.7
Unknown	9 (12.7)	4 (10.0)	5 (16.1)	0.57	2.0	0.2	22.1
Hepatic encephalopathy	3 (1 - 3)	3 (1 - 3)	3 (1 - 3)	0.90	1.0	0.7	1.4
Hemoglobin (g/dL)	9.3 (8.2 - 10.9)	9.6 (8.5 - 10.9)	8.9 (8.0 - 11.0)	0.27	1.0	1.0	1.1
Leucocytes (103/µl)	10.3 (5.5 - 15.9)	11.3 (5.0 - 16.3)	10.1 (5.5 - 15.9)	0.27	1.0	1.0	1.0
Platelets (103/µl)	92 (50 - 134)	86 (43 - 120)	97 (58 - 156)	0.91	1.0	1.0	1.0
Ammonia (µmol/L) (n = 46)	106 (81 - 180)	107 (79 - 192)	100 (77 - 160)	0.43	1.0	1.0	1.0
International normalized ratio	2.0 (1.7 - 2.4)	2.2 (1.8 - 2.7)	1.8 (1.5 - 2.3)	0.09	1.2	1.0	1.5
Bilirubin (mg/dL)	5.4 (2.4 - 21.1)	9.2 (2.9 - 22.1)	4.0 (1.7 - 13.3)	0.13	1.0	1.0	1.1
Albumin (g/dL)	24.9 (20.7 - 31.4)	23.7 (19.5 - 30.9)	26.4 (21.0 - 31.4)	0.51	1.0	1.0	1.0
Creatinine (mg/dL)	1.50 (0.83 - 2.40)	1.53 (0.90 - 2.55)	1.14 (0.77 - 2.20)	0.49	1.1	0.9	1.3
Sodium (mmol/L)	135 (129 - 140)	134 (125 - 139)	136 (132 - 142)	0.35	1.0	1.0	1.0
C-reactive protein (mg/L) (n = 69)	34.3 (18.4 - 62.5)	37.2 (23.6 - 72.0)	20.3 (12.6 - 51.1)	0.04	1.0	1.0	1.0
Lactate (mmol/L) (n = 63)	2.3 (1.5 - 3.6)	2.7 (1.8 - 4.4)	1.9 (1.4 - 2.7)	0.03	1.2	1.0	1.4
Renal replacement therapy	8 (11.3)	3 (7.5)	5 (16.1)	0.17	1.4	0.9	2.3
Vasopressors	37 (52.1)	23 (57.5)	14 (45.2)	1.00	1.0	0.5	2.0
Invasive mechanical ventilation	18 (25.4)	12 (30.0)	6 (19.4)	0.68	1.1	0.7	1.9
PaO_2_/FiO_2_	300 (218 - 382)	283 (157 - 390)	316 (222 - 381)	0.57	1.0	1.0	1.0
CLIF-SOFA	13 (11 - 15)	14 (12 - 16)	11 (9 - 14)	0.10	1.0	1.0	1.1
Child-Pugh	12 (10 - 13)	12 (11 - 13)	11 (9 - 12)	0.17	1.0	1.0	1.1
MELD	27 (20 - 32)	28 (23 - 35)	26 (17 - 31)	0.10	1.0	1.0	1.0
ACLF (grade)*	2 (1 - 3)	3 (2 - 3)	2 (1 - 3)	0.04	1.7	1.0	2.7
Organ failures*	2 (1 - 3)	3 (2 - 3)	2 (1 - 3)	0.01	2.0	1.2	3.4
APACHE II	21 (16 - 23)	21 (17 - 25)	20 (16 - 23)	0.14	1.0	1.0	1.0

OR - odds ratio; 95%CI - 95% confidence interval; PaO_2_/FiO_2_ - arterial oxygen partial pressure with oxygen inspiration fraction ratio; CLIF-SOFA - Chronic Liver Failure-Sequential Organ Failure Assessment; MELD - Model for End-Stage Liver Disease; ACLF - Acute-on-Chronic Liver Failure Score; APACHE II - Acute Physiology and Chronic Health Evaluation II.

* Definitions of organ failures based on Chronic Liver Failure-Sequential Organ Failure Assessment. Results expressed as n (%) or median (inter-quartile range).

**Table 2:** Acute-on-chronic liver failure patient characteristics at day 3 after intensive care unit admission and univariate association with 28-day mortality

	Overall ^†^(n = 66)	Dead at 28 days(n = 35)	Alive at 28 days(n = 31)	p value	OR	OR 95%CI	
						Inferior	Superior
Hepatic encephalopathy	2 (1 - 3)	3 (2 - 4)	2 (1 - 2)	0.03	1.5	1.0	2.1
Hemoglobin (g/dL)	8.5 (7.6 - 10.0)	8.8 (7.7 - 10.0)	8.4 (7.4 - 10)	0.90	1.0	0.8	1.3
Leucocytes (103/*µ*l)	11.0 (5.4 - 13.7)	11.9 (7.5 - 17.1)	6.6 (5.0 - 12.7)	0.09	1.0	1.0	1.0
Platelets (103/*µ*l)	63 (40 - 105)	46 (38.5 - 94)	68 (50 - 110)	0.23	1.0	1.0	1.0
Ammonia (*µ*mol/L) (n = 46)	83 (62 - 107)	74 (57 - 107)	99 (76 - 106)	0.44	1.0	1.0	1.0
International normalized ratio	2.1 (1.6 - 2.8)	2.5 (1.9 - 3.3)	1.7 (1.4 - 2.0)	0.002	3.7	1.6	8.5
Bilirubin (mg/dL)	6.6 (2.1 - 19.7)	12.4 (3.2 - 21.9)	3.9 (1.4 - 14.3)	0.07	1.0	1.0	1.1
Creatinine (mg/dL)	1.0 (0.8 - 2.3)	1.0 (0.8 - 2.3)	0.9 (0.7 - 1.8)	0.63	1.1	0.7	1.7
Sodium (mmol/L)	137 (133 - 142)	135 (132 - 142)	140 (135 - 143)	0.09	0.9	0.9	1.0
C-reactive protein (mg/dL) (n = 64)	49 (28 - 77)	57 (31 - 90)	36 (25 - 63)	0.05	1.0	1.0	1.0
Lactate (mmol/L) (n = 61)	2.0 (1.4 - 3.3)	3.1 (1.8 - 5.3)	1.4 (1.1 - 2.0)	0.002	4.7	1.8	12.6
Renal replacement therapy	16 (23)	12 (75)	4 (25)	0.10	0.3	0.1	1.2
Vasopressors	37 (52)	26 (70.3)	11 (29.7)	0.02	0.3	0.1	0.8
Invasive mechanical ventilation	23 (32)	18 (78.3)	5 (21.7)	0.01	0.2	0.1	0.7
PaO_2_/FiO_2_	263 (201 - 363)	230 (144 - 364)	300 (237 - 360)	0.04	1.0	1.0	1.0
CLIF-SOFA	13 (9 - 17)	15 (11 - 19)	9 (8 - 13)	< 0.001	1.3	1.1	1.5
Child-Pugh	12 (10 - 13)	13 (11 - 13)	10 (9 - 12)	< 0.001	1.7	1.2	2.3
MELD	26 (21 - 35)	31 (25 - 38)	25 (15 - 29)	0.003	1.1	1.0	1.2
ACLF (grade)*	2 (1 - 3)	3 (2 - 3)	1 (0 - 2)	< 0.001	3.4	1.9	6.2
Organ failures*	2 (1 - 3)	3 (2 - 4)	1 (1 - 2)	< 0.001	2.6	1.6	4.3
Delta CLIF-SOFA	0 (-2 - 3)	1 (-1 - 5)	-1 (-3 - 1)	0.004	1.3	1.1	1.5

PaO_2_/FiO_2_ - arterial oxygen partial pressure with oxygen inspiration fraction ratio; CLIF-SOFA - chronic liver failure-sequential organ failure assessment; MELD - model for end-stage liver disease score; MELD - model for end-stage liver disease score; ACLF - acute-on-chronic liver failure score.

* Definitions of organ failures based on CLIF-SOFA.

† Five patients were dead by day 3 post ICU admission thus n = 66. Results expressed as n (%) or median (inter-quartile range).

There were 71 ACLF patients with a median age of 59 (51 - 64) years; 81.7% were male. Alcohol consumption alone (53.5%) or combined with hepatitis C virus infection (11.3%) were the most frequent etiologies of cirrhosis, and the most common precipitating events of acute illness were infection (53.5%) and gastrointestinal bleeding (22.5%).

The median clinical severity scores at ICU admission were APACHE II, 21 (16 - 23); ACLF grade, 2 (1 - 3); CLIF-SOFA, 13 (11 - 15); Child-Pugh, 12 (10 - 13); and MELD 27 (20 - 32).

The ACLF severity scores at ICU admission were as follows: no-ACLF, 11.3%; grade one, 14.1%; grade two 28.2%; and grade three 46.5%. The proportion of organ failure at ICU admission was one: 4.2%; two: 42.3%; three: 32.4% four: 16.9% and five: 4.2%.

The day 3 analysis excluded 5 patients who had died (n = 66) and the median ACLF grade was 2 (1 - 3), the median CLIF-SOFA score was 13 (9 - 17) with a Delta CLIF-SOFA of 0 (-2 - 3); the median Child-Pugh score was 12 (10 - 13) and the median MELD score was 26 (21 - 35). The acute-on-chronic liver failure severity scores at day 3 were as follows: no-ACLF, 16.7%; grade one, 18.2%; grade two, 27.3% and grade three, 37.9%. The proportion of organ failure at day 3 was zero, 12.7%; one, 19.7%; two, 25.4%; three, 18.3%; four, 12.7%; five, 5.6%; and six, 5.6%.

**Figure 1:**
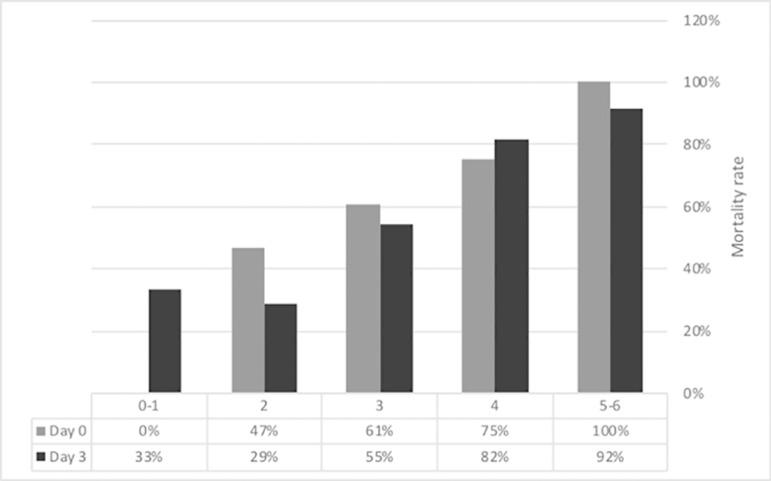
Number of failing organs in acute-on-chronic liver failure patients (N0 = 71, N 3= 66) and the associated 28-day mortality rate.

**Figure 2:**
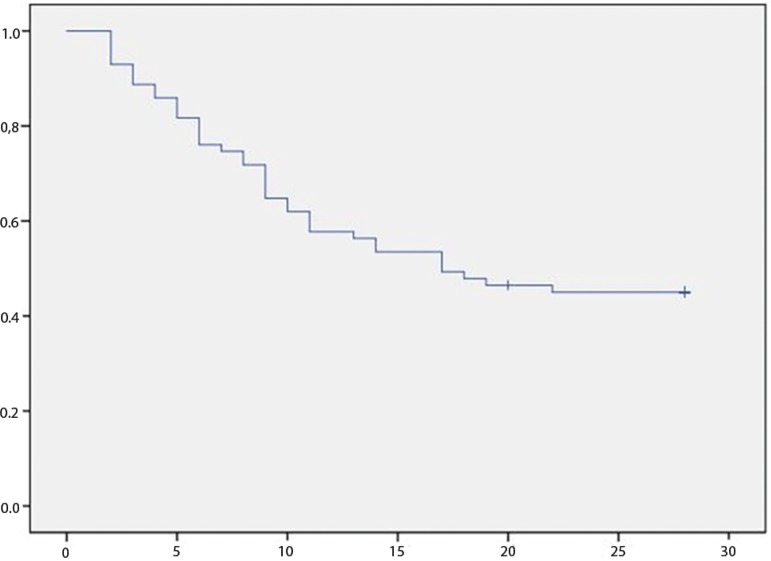
Kaplan-Meier 28-day survival curve for acute-on-chronic liver failure patients (N = 71) admitted to the intensive care uni

The 28-day mortality rate post-ICU admission was 56.3%, and the in-ICU mortality rate was 49.3%. Liver transplantation was performed on 11 patients (15.5%) with a 36.4% 28-day mortality rate. The main causes of death were sepsis (54.7%) and bleeding (18.9%). At ICU admission, the mortality rates by ACLF score were one, 30.0%; two, 45.0%; and three, 72.7%. Organ failure mortality rates at ICU admission and day 3 are shown in figure 1. The Kaplan-Meier survival curve is depicted in figure 2.

### Risk factor analysis for 28-day mortality rate

Among the ICU admission (day 0) variables, a significant univariate association with 28-day mortality was found for bleeding as the precipitating event, INR, C-reactive protein, arterial blood lactate, CLIF-SOFA, MELD, ACLF grade and organ failure number (Table 1). Bleeding was transformed into a dichotomous variable, and it was significantly associated with lower mortality (p = 0.096, odds ratio - OR 0.37; 95% confidence interval - 95%CI 0.12 - 1.2); bleeding was included in the multivariate analysis. the multivariate logistic regression (n = 61) for ICU admission variables found that organ failure number (p = 0.02; OR 2.1; 95%CI 1.2 - 3.9) was the only independent risk factor for 28-day mortality.

Day 3 univariate associations with 28-day mortality found statistical significance for hepatic encephalopathy, INR, C-reactive protein, arterial blood lactate, renal replacement therapy, vasopressors, invasive mechanical ventilation, PaO2/FiO2 ratio, all clinical severity scores (CLIF-SOFA, Child-Pugh, MELD, ACLF grade), organ failure number and Delta CLIF-SOFA (Table 2).

The multivariate logistic regression (n = 55) found day 3 arterial blood lactate concentration (p = 0.02; OR 6.3; 95%CI 1.4 - 28.6) and INR (p = 0.03; OR 10.2; 95%CI 1.3 - 82.8) to be independent risk factors. Interestingly, an increasing value of Delta CLIF-SOFA (p = 0.06; OR 0,5; 95%CI 0.2 - 1,0) presented a strong trend towards a significant association with increased mortality, as would be expected in patients with deteriorating clinical condition by day 3.

Patients submitted to liver transplant (n = 11) within the initial 28 days of ICU admission did not have a significantly different 28-day mortality rate (p = 0.19; OR 0.41; 95%CI 0.11 - 1.5) in this cohort of patients, although the observed survival rate of 63.6% in this subset of patients is clinically valuable.

No survivors presented with a CLIF-SOFA ≥ 21, Child-Pugh ≥ 14, MELD ≥ 42, organ failure ≥ 5 or lactate concentration ≥ 3.2mmol/L at the day 3 assessment.

## DISCUSSION

Our study analyzed the results of a 3-year cohort of ACLF patients admitted to a general ICU in a regional reference center for LT. The typical patient was a 60-year-old male with alcoholic liver cirrhosis presenting with septic shock, multiorgan failure and ACLF grade 3 at ICU admission.

When comparing our results with reference studies in the literature,^(6,10)^ our study presented patients with overall higher clinical severity and higher mortality rates.

Weil et al. conducted a recent large meta-analysis of 1904 cirrhotic patients, including 369 ACLF patients admitted to intensive care. Interestingly, the presence of ACLF criteria appeared to have had no significant impact on ICU mortality in this meta-analysis, although the authors admit limitations to this observation.^(10)^ In this work, the typical cirrhotic patient was male, under 60 years old, with alcohol-related cirrhosis, and the primary reason for ICU admission was variceal bleeding. The proportions of organ failure ≥ 2 and organ failure ≥ 4 were 47.7% and 15.6%, respectively, and the overall in-ICU mortality rate was 42.4%. Altogether, both the proportions of organ failure and the mortality rates were lower than those in our study. One of the findings of this study was that patients with variceal bleeding showed significantly lower mortality than those with infection as the ACLF precipitating event.^(10)^ This was not the case in our study, where infection was the most frequent precipitating ACLF event and may have influenced the observed increase in mortality.

In a seminal study, Moreau et al. reported the outcomes in 415 ACLF patients and presented a definition for this syndrome.^(6)^ Acute-on-chronic liver failure was present in 303 patients at study enrollment, and importantly, this study included 208 ACLF patients who did not receive intensive care, which may have had an influence on the clinical results. The precipitating events included bacterial infection (32.6%), gastrointestinal hemorrhage (13.2%), and unidentified causes (43.6%). The main causes of death were multiorgan failure without shock and both septic and hypovolemic shock types.^(6)^ The proportion of ACLF grade one was 48.8%, grade two was 36.6%, and grade three was 15.5% with mortality rates of 23.3%, 31,3% and 74,5%, respectively.^(6)^ Overall, these results described less severe patients and presented lower mortality rates than in our cohort.

Both of these reference studies reported less severe patients and lower mortality rates than our study, although clinical result comparisons should be interpreted cautiously due to the large heterogeneity of the ACLF patients studied, including heterogeneity in clinical severity, clinical settings and primary precipitating events. Subgroup analyses, including precipitating events, should provide more homogenous groups for future studies. Furthermore, our institution serves as a regional reference center for LT, and we treat ACLF patients in severe condition transferred from other acute care hospitals. This fact may help to explain and understand the increased burden of severity and mortality we report in our study.

We identified organ failure as the single independent risk factor for 28-day mortality at ICU admission in our cohort of patients. This finding was further explored by our colleagues Cardoso et al. in a recently published multicenter study of mortality prediction in ACLF patients in the ICU. In this study, a training set of 240 patients was used to derive the “LacOF” model using organ failure and arterial blood lactate concentration at ICU admission. This model’s mortality prediction results (area under the curve - AUC, 0.85) outperformed the CLIF-SOFA, CLIF-ACLF, MELD and APACHE II scores in a validation set of 237 patients.^(18)^ This stresses the clinical importance of discriminating the exact extent (number of failing organs) of multiorgan failure at ICU admission, whereas the ACLF grading score does not discriminate patients with three or more organ failures (all included in ACLF grade 3).

Our day 3 analysis revealed that lactate concentration and INR were independent risk factors, both acting as surrogate markers of persistent hepatic dysfunction and failure to recover from the initial ACLF decompensation insult. Lactate undergoes hepatic clearance, and its concentration increases with liver failure and anaerobic stress conditions such as sepsis and shock. These conditions were highly frequent among our patient cohort. Arterial blood lactate concentration is widely used as a surrogate marker for the general severity of critical illness, and prognostic scores for critically ill cirrhotic patients have incorporated this variable into their scoring algorithms. These include the SOFA-lactate,^(19)^ Child-Pugh + lactate,^(20,21)^ albumin-bilirubin-clotting (ABC) + lactate^(22)^ and Royal Free Hospital (RFH) algorithms.^(23)^ Furthermore, the INR value, the prothrombin time ratio, is a valuable biomarker of hepatic synthesis function that is specifically used in clinical practice to assess the degree of hepatic dysfunction and coagulopathy; it is used as a liver transplant criterion^(24)^ and in the CLIF-SOFA score. In our study, both lactate concentration and INR proved to be clinically useful for clinical severity assessment in ACLF patients, providing a prognostic indication at day 3 of ICU for decisions regarding vital therapy.

Liver transplantation in our cohort of ACLF patients did not achieve an association with 28-day mortality, possibly due to an underpowered sample size. Nonetheless, it is a vital therapeutic measure for certain patients, and we believe our results clearly favor the decision to transplant well-selected patients. This has been demonstrated by Artru et al. in his work with 73 ACLF grade 3 patients receiving liver transplant while in the ICU with a clear one-year survival benefit when compared to nontransplanted controls (83.9 *versus* 7.9%, p < 0.0001).^(3)^ The observed high survival rate did not significantly differ from liver transplant matched controls with a lower grade of ACLF. The authors of that study emphasize the notion of a “transplantation window” where these severe patients could benefit from a rapid liver transplant decision process after achieving initial ICU treatment and stabilization for their ACLF precipitating event.^(3)^

Finally, the severity scores and their prognostic value throughout the first week of the ICU stay are well described. Whether clinical scores should serve as a rationale to determine the benefit of supportive therapy or discontinuation of intensive care due to futility, as suggested by Gustot et al. (when organ failure ≥ 4 at days 3 - 7), is a matter of debate.^(7,12,13)^ In our cohort, two patients with day 3 organ failure 4 were alive 28 days after ICU admission, and both had a hospital discharge home. Clinical severity scores provide a basis for analysis but should not replace clinical judgment.^(9,25,26)^Decisions regarding vital support and liver transplant in critical patients remain complex and should include and respect the patients, families, and healthcare teams when setting goals of care and expectations.

Limitations in our study include the retrospective characteristic of the study and the relatively low number of patients, precluding a deeper analysis of mortality risk factors. Furthermore, heterogeneity of the ACLF patients’ clinical settings, precipitating events, clinical severity and outcomes reporting in the literature prevented a clear, direct comparison of results.

## CONCLUSION

The typical patient in our study was a 60-year-old male with alcoholic liver disease admitted to the intensive care unit for septic shock and acute-on-chronic liver failure grade 3 multiorgan failure.

Our results presented overall higher clinical severity and mortality rates when compared to the literature, and in our study, the number of organ failures on intensive care unit admission and the arterial blood lactate concentration and international normalized ratio on day 3 were independent risk factors for 28-day mortality.

We consider it essential that acute-on-chronic liver failure patients be treated in the intensive care unit and that a timely clinical decision is vital for liver transplant therapy in well-selected patients.
